# The Therapeutic Response of Gastrointestinal Stromal Tumors to Imatinib Treatment Assessed by Intravoxel Incoherent Motion Diffusion-Weighted Magnetic Resonance Imaging with Histopathological Correlation

**DOI:** 10.1371/journal.pone.0167720

**Published:** 2016-12-02

**Authors:** Feng Pan, Jie Den, Chunfang Zhang, He Wang, Jin Cheng, Weizhen Wu, Nan Hong, Yi Wang

**Affiliations:** 1 Department of Radiology, People’s Hospital, Peking University, Beijing, China; 2 Department of Medical Imaging, Ann & Robert H. Lurie Children's Hospital of Chicago, Department of Radiology, Feinberg School of Medicine, Northwestern University, Chicago, Illinois, United States of America; 3 People’s Hospital, Peking University, Clinical Epidemiology and Medical Statistics, Beijing, China; 4 GE Healthcare, Shanghai, China; Northwestern University Feinberg School of Medicine, UNITED STATES

## Abstract

**Purpose:**

To exploit the intravoxel incoherent motion (IVIM) diffusion-weighted (DW) MRI when evaluating the therapeutic response of gastrointestinal stromal tumors (GIST) to Imatinib in a mouse model.

**Materials and Methods:**

Mice with xenografts bearing cells from the GIST-T1 cell line were randomly divided into a treated group receiving Imatinib and a control group. DWMRI scans with 14 b-values (0–1500 s/mm^2^) were performed before and after treatment (days 1, 3 and 7). IVIM related parameters perfusion fractions (*f*p) and perfusion-related diffusion coefficients (D*) and the conventional apparent diffusion coefficients (ADC) were calculated by fitting the DWMRI signal decay. The mean changes from baseline to each post-treatment time point for each measurement (ΔADC, Δ*f*p and ΔD*) were calculated. The differences of mean changes between the two groups were tested for statistical significance. Histopathological analyses including Ki-67, CD31, TUNEL and H&E were conducted in conjunction with the MRI scans.

**Results:**

Increases in ADC of the treated group were higher than those of the control group after treatment, whereas statistical significances were not observed. Compared to the control group, D* in the treated group decreased significantly (ΔD*_treated_ = -41%, -49%, and -49% with *P* = 0.0001, 0.0001 and 0.0001), and *f*p increased significantly (Δ*f*p_treated_ = 79%, 82% and 110%, with *P* = 0.001, 0.0001 and *P* = 0.0007) on days 1, 3 and 7 after treatment. Histopathological analyses demonstrated different tumor tissue characteristics between the treated and control groups.

**Conclusion:**

IVIM measurements may serve as more sensitive imaging biomarkers than ADC when assessing GIST response to Imatinib as early as one day after treatment.

## Introduction

Gastrointestinal stromal tumors (GIST) are the most common mesenchymal cancer of the gastrointestinal tract. They originate from the proliferation of interstitial cells of Cajal[1]. The cells in GIST express a growth factor receptor with tyrosine kinase activity termed c-kit. This receptor, the product of the proto-oncogene c-kit, can be detected by immunohistochemical staining for CD117[1]. Imatinib mesylate (STI571) is used as the first-line treatment of advanced GIST[1, 2]. However, primary Imatinib resistance is detected in approximately 15% of GISTs, and secondary resistance to Imatinib is developed in most responders[3, 4]. Additionally dose-dependent adverse effects associated with Imatinib have occurred in patients with metastatic or surgically unresectable GISTs[5]. In order to optimize individual patient care and to avoid ineffective treatment and unnecessary toxicity, several noninvasive imaging modalities have been used to evaluate GIST response to Imatinib[6–8].

Monitoring tumor size using computed tomography (CT) has been accepted as the standard method for assessing GIST response to Imatinib in accordance with the Response Evaluation Criteria In Solid Tumors (RECIST)[9]. Alternatively, using a combination of a modified tumor size measurement (>10% change) and a measurement of tumor density (>15% change), shown as attenuation in CT, is promising for evaluating GIST response to Imatinib[10, 11]. Reduction in tumor density in conjunction with tumor size has been shown to be more strongly correlated with tumor metabolic response, as measured by ^18^F-Fluoro-2-deoxyglucose (^18^F-FDG) positron emission tomography (PET)/CT, than tumor size alone[11]. ^18^F-FDG PET/CT can also be used to monitor tumor response as it can detect functional GIST tumor metabolism changes as early as 24 hours after the initial administration of Imatinib treatment[10, 12, 13]. However, approximately 20% of untreated and malignant GISTs cannot be detected by ^18^F-FDG PET/CT. In addition, ^18^F-FDG PET/CT's high costs and radiation exposure do not make it an ideal option for routine treatment response evaluations[14].

Diffusion-weighted magnetic resonance imaging (DWMRI) is a non-invasive and nonionizing radiation imaging method that can measure tissue water mobility changes in order to evaluate GIST response to Imatinib[8, 15, 16]. In conventional DWMRI, tissue water mobility can be quantified with the apparent diffusion coefficient (ADC), which is calculated as the slope of the logarithm signal decay on diffusion-weighted images acquired at two or three diffusion weightings (e.g. b-value = 0–1000 sec/mm^2^). However, ADC measurements generally do not distinguish between multi-compartmental (e.g. diffusion *vs*. perfusion and intracellular *vs*. extracellular) effects in biological tissues[17]. More advanced diffusion models based on intravoxel incoherent motion (IVIM) phenomena have been developed to separate diffusion properties in different compartments. IVIM measurements of the pure diffusion coefficient (D), fast (pseudo-perfusion) diffusion coefficient (D*), and fast fractional volume (*f*p)[17, 18] have been exploited to differentiate between benign and malignant tumors[19–21] and assess therapeutic responses to chemotherapy in different tumor types[22–25].

To our knowledge, little work has been done using histopathologic validation to evaluate the ability of IVIM DWMRI measurements to assess GIST response to Imatinib. The purpose of this study was to evaluate the therapeutic response of GIST to Imatinib treatment on days 1, 3, and 7 post-treatment using IVIM DWMRI, and to compare imaging findings with histopathological changes secondary to treatment.

## Materials and Methods

### Animal Model

This study was approved by the Animal Experimental Ethical Committee of the People's Hospital of Peking University and was conducted in accordance with their guidelines. BALB/c nude female mice, which weighed between 18 and 23 g and ranged in age from 6 to 8 weeks old, were obtained from Vital River Laboratories (Beijing, China). They were acclimatized for one week and then caged in a pathogen-free facility in accordance with the animal welfare guidelines set by the Office of Laboratory Animal Welfare (OLAW) of Peking University People’s Hospital. They were housed under the following conditions: temperature of 22°C, humidity of 61%, and 12 hour light–dark rhythm; they were provided with an ordinary diet and purified water. They were observed by veterinarians three times a day and were treated using the prescribed plan of action in case of illness, e.g., initiation of treatment and/or euthanasia.

They were implanted with GIST xenografts (described in Tumor Model section) and randomized into a treated group and a control group. All mice underwent MRI exams before treatment (day -1) and on days 1, 3, and 7 after treatment. All mice were euthanized by decapitation under anesthesia on day 7 after their last MRI scan; at that time tumors were resected and prepared for gross anatomic and histopathological analyses. Humane endpoints of mice included weight loss of 20%-25%, loss of appetite (complete anorexia for 24 hours), weakness preventing them from obtaining food or water, moribund state, infection, unable to participate in normal activities due to the tumors, having a tumor larger than 20mm, or severe ulceration.

### Tumor Model

Cells from the GIST-T1 cell line with a mutation on KIT exon 11 were purchased from Cell Signaling Technical (Beverly, MA, USA). The cells were maintained in Dulbecco’s modified Eagle’s medium (DMEM) and supplemented with 10% fetal bovine serum (FBS), 100 units/ml Penicillin, and 100 units/ml Streptomycin. They were kept at 37°C with a 95% O_2_ concentration and 5% CO_2_ concentration. They were passaged twice per week with a 1:2 split using 0.25% trypsin (HyClone, Ft. Collins, CO, USA). DNA from GIST-T1 cells was analyzed using the Profiler Plus kit (CHGB, Beijing, China). The cells' DNA profile was compared with data from the American Type Culture Collection to ensure authenticity.

To establish a GIST xenograft, approximately 2.5 × 10^6^ cells in a volume of 100μL phosphate buffered saline (PBS) were subcutaneously implanted into the left and right flanks of each mouse under anesthesia. Xenograft tumors were measured daily using a caliper until tumor volumes reached 200 to 350 mm^3^. When all tumors reached an acceptable volume, the following treatment protocol was implemented.

### Treatment Protocol

Imatinib (STI571, Methanesulfonate Salt, >99%) was purchased from GenDEPOT (Barker, TX, USA). Mice in the treated group were administered Imatinib (powder dissolved into normal saline) orally by gavage twice per day (150 mg/kg) for 7 consecutive days starting on day 0. The mice in the control group were administrated placebos (normal saline) at the same time as the treated group.

### Imaging Acquisition

MRI scans were performed on a 3.0 Tesla MRI scanner (Discovery 750, GE Healthcare, Waukesha, WI, USA). Each mouse was anesthetized by intraperitoneal injection of pentobarbital sodium (50mg/kg, intraperitoneal) and then placed in a test tube filled with salt alginate impression gel, which had been dissolved in warm water to mitigate the susceptibility artifacts arising from the tissue-air interface at the subcutaneous xenograft tumor areas and to maintain the mouse's body temperature. The tube containing the mouse was then placed in a small animal birdcage coil (Magtron Inc., Jiangyin, China) and kept in the supine position during MR imaging. Anatomic MR images for tumor volume measurements were acquired using axial, sagittal and coronal T2-weighted (T2W) spin-echo (SE) sequence with the following parameters: TR/TE = 2800/72msec; bandwidth = 31.5kHz; acquisition matrix = 256×192; field of view (FOV) = 70×35mm^2^; section thickness/gap = 1.5/0.5mm and number of excitation (NEX) = 4.

DWMR images were acquired using a single-shot spin-echo echo-planar imaging (DW-SE-EPI) sequence with a spatial-spectral excitation pulse to suppress fat signals. Parallel imaging using the array spatial-sensitivity encoding technique (ASSET) with an acceleration factor of 2 was employed to reduce the echo train length and thus mitigate image distortion. A wide range of diffusion weighting with 14 b-values (0, 10, 20, 30, 50, 80, 130, 200, 300, 400, 600, 800, 1000 and1500 sec/mm^2^) in three orthogonal gradient directions (x, y, and z) was applied to detect isotropic water mobility. Other imaging parameters included: TR/TE = 2500/42 msec; matrix = 64×64; FOV = 70×35mm^2^; section thickness/gap = 1.5/0.5 mm; NEX was different for each b value (NEX = 1 for b-value = 0–300, NEX = 2 for b-value = 400–600, and NEX = 4 for b-value = 800–1500 sec/mm^2^); partial Fourier factor = 0.625. The acquisition time for multi-b values of DW MR imaging was 4 minutes for each mouse. The total acquisition time for anatomic and DW MR imaging was about 15 minutes total for each mouse.

### Post-processing

Anatomic and DWMR images were transferred to a workstation (SW45; GE Healthcare, Waukesha, WI, USA). One radiologist, with 3 years of experience in MRI interpretation, did the tumor measurements, blinded to treatment conditions and histopathology results. Three-dimensional maximal diameters of each tumor were measured on the axial, coronal and sagittal T2W MR images, and the tumor volume (T_v_) was calculated as 4/3π × (length × width × height)/2.

On each DW image slice with b-value = 0 sec/mm^2^, a region of interest (ROI) was manually drawn along the margin of the entire tumor and automatically copied onto the DW images with all other b values. Within each ROI, the averaged signal intensity at each b-value was calculated. In standard clinical protocol, conventional ADC was calculated by the linear fitting of the logarithm of signal intensities at b-value = 0 and 1000 sec/mm^2^.

IVIM parameters, including the fast diffusion coefficient (D*) and the corresponding fractional volume (V_fast_), slow diffusion coefficient (D) and the corresponding fractional volume (V_slow_), were calculated based on the bi-exponential model *S*(*b*)/*S*(0) = *V*_*slow*_ × *e*^−*bD*^ + *V*_*fast*_ × *e*^−*b*(*D*+D*)^ where V_fast_+V_slow_~1, and S(b) and S(0) represented signal intensity at each corresponding b-value. The trust-region-reflective non-linear fitting algorithm (Matlab, MathWorks, Inc., Natick, MA, USA) was used for bi-exponential signal decay fitting to derive all four parameters. Prior to the non-linear fitting, the initial guesses for D and V_slow_ were estimated by a linear fitting of DW signals at higher b-values (>200 sec/mm^2^), where fast diffusion effects can be ignored. The initial guess of D* was estimated by a linear fitting of DW signals at lower b-values (<80 sec/mm^2^), where fast diffusion effects dominate the signal decay. Finally, the fractional volume of the fast component that is presumably considered as pseudo-perfusion fraction (*f*p) was calculated as 100%×V_fast_/(V_fast_+V_slow_). In order to compare the fitting behavior using the two-compartment bi-exponential IVIM and mono-exponential signal decay model, both methods were performed to fit the signal decay with the full range of 16 b-values in a subset of animals.

### Histopathological Analyses

After imaging acquisition on day 7 after treatment, mice from both the treated and control groups were euthanized. The entire tumor tissue was resected, fixed in 10% formalin for 24 to 48 hours, and immersed in 70% ethanol. Tumor specimens were paraffin-embedded and sectioned at a 5-μm thickness. Tissue sections were stained with immunohistochemistry on the Ventana Discovery XT Autostainer (Ventana, Tucson, AZ). Immunohistochemistry analyses including Ki67, terminal deoxynucleotidyl tranferase mediated dUTP nick end labeling (TUNEL), and CD31 staining were performed in areas containing viable tumor tissues. Tumor cells sufficiently stained with chromogen were considered to be positive compared with the surrounding tissues. For each type of histopathological analysis, each staining slide was digitized with an optical magnification (×200 or ×400) using the LEICA DFC 550 Digital Microscope Camera (Wetzlar, Germany). One pathologist with 10 years of experience defined the viable tumor areas by avoiding significant necrotic areas on the microscopic image slides. The microscopic images were then analyzed in Image J (version 1.42; National Institutes of Health, Bethesda, MD) to automatically differentiate target cells with positive staining and background cells; then the detected target cells were manually confirmed based on the image intensity and minimum particle size threshold. At least three sections from each tumor were measured and averaged.

For the Ki67 staining (ab15580; Abcam, Cambridge Science Park, UK), the proliferating cell density was calculated as the ratio of Ki-67 positive cells to the total number of viable cells. In TUNEL staining (Roche, Basel, Switzerland), the apoptotic cell density was calculated as the ratio of cells with positive TUNEL expression to the total number of viable tumor cells. In CD31 staining (sc1506; Santa Cruz Biotechnology, Santa Cruz, CA), microvessel endothelial cells were identified, and the microvessel density (MVD) was calculated as the total number of vessels divided by the total number of viable tumor cells. Hematoxylin and eosin (H&E, Sigma-Aldrich; Ventana, Tucson, AZ) was performed to delineate tumor necrosis, and the necrotic fraction was calculated as the ratio of the necrotic area to the total tumor area.

### Statistical Analysis

The median and interquartile ranges of tumor volume and parameters derived from DWMRI were calculated because data distribution showed substantial departure from normality (standardized kurtosis and standardized skewness >2 and significant Shapiro-Wilks test for normality, *P*<0.0001).

The median percentage change of each MRI measurement in comparison to the baseline (i.e. pre-treatment) for each tumor was calculated (ΔTv%, ΔADC%, ΔD*% and Δ*f*p%, ΔD%). The logarithmic transformation was performed to reach the precondition of the normal distribution for the mixed linear model analysis. After logarithm transformation, ΔTv%, ΔADC%, ΔD*%, Δ*f*p% and ΔD% were compared between the control group and treated group using the mixed linear regression model.

Spearman correlation analyses were performed between each IVIM measurement (i.e. ADC, D*, *f*p and D) measured on day 1, 3 and 7 post-treatment and each pathological analysis (i.e. Ki-67 (%)-proliferating cell density, Tunel (%)-apoptotic cell density, CD31 (%)-microvessel endothelial cell density and HE (%)-necrotic fraction). In addition, Mann-Whitney U test was used to compare each pathological value between treated and control group.

P-values below 0.05 were considered statistically significant. All analyses were performed using the statistical software Stata (version13.0; Stata Corp LP, College Station, Texas, USA).

## Results

Thirteen mice were initially randomized into the treated group (n = 8) and the control group (n = 5). One mouse in the control group was excluded because of severe ulcerations on day 3 after treatment and was euthanized. One mouse in the treated group was excluded because of severe image distortion and chemical shift artifacts on the DW images (the mouse was too weak to undergo anesthesia for its MR scan and was euthanized on day 7 with other mice). A total of 7 mice with 14 tumors were ultimately included in the treated group, and 4 mice with 8 tumors were included in the control group.

**Fig 1:**
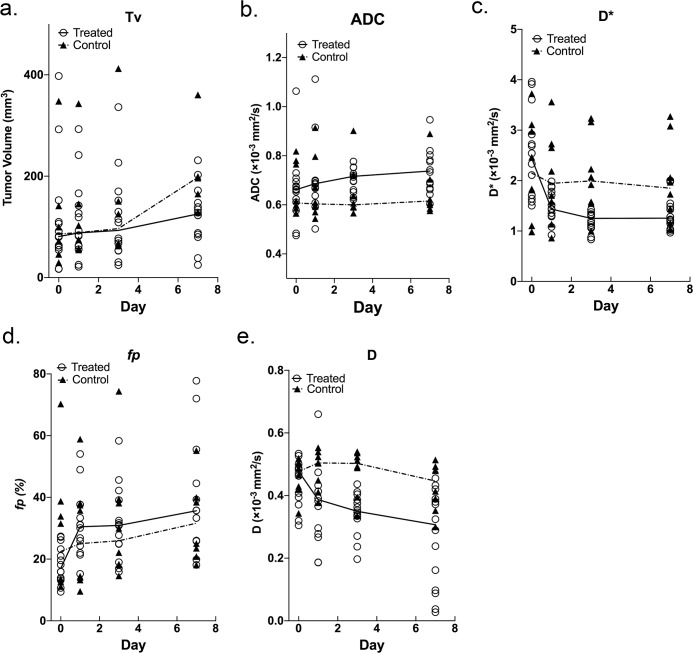
Individual data and median values at each time point for control and treated group. Individual replicates with median connected plots show (**a**) Tv (tumor volume), (**b**) ADC (apparent diffusion coefficient), (**c**) D* (fast diffusion coefficient), (**d**) *f*p (fast fractional volume), (**e**) D (slow diffusion coefficient) of the treatment group and control group. Individual data are presented as dots. The lines represent median value.

**Fig 2:**
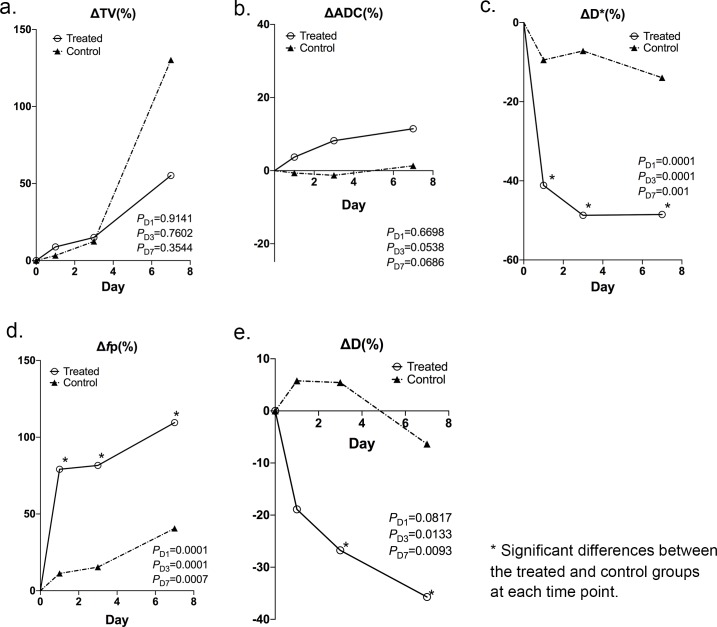
Median percentage changes at each time point for control and treated group. Median percentage changes at each time point (days 1, 3 and 7) compared with baseline for (**a**) ΔTv (tumor volume), (**b**) ΔADC (apparent diffusion coefficient), (**c**) ΔD* (fast diffusion coefficient), (**d**) Δ*f*p (fast fractional volume), (**e**) ΔD (slow diffusion coefficient) of all mice in the treated group (solid line) and control group (dashed line), respectively.

Tumor volume and each of the diffusion measurements at each time point were shown in **Table 1** with median and interquartile values and (**Fig 1)** showing median values. For both groups, the median percentage post-treatment change of these parameters on days 1, 3 and 7 compared with the pre-treatment baseline were listed in **Table 2** and (**Fig 2)**.

**Table 1:** Tumor MRI measurements before and after treatment.

Median (Interquartile range)	D0	D1	D3	D7
**Tv (mm**^**3**^**)**				
Treated (N = 14)	80.95	88.09	93.03	125.58
	(8.09–120.52)	(60.39–148.05)	(53.52–156.50)	(83.72–153.56)
Control (N = 8)	85.94	88.76	96.60	197.84
	(41.85–193.19)	(56.74–194.73)	(64.25–216.03)	(155.99–449.40)
**ADC (×10**^**−3**^ **mm**^**2**^**/s)**				
Treated (N = 14)	0.66	0.69	0.72	0.74
	(0.60–0.70)	(0.63–0.70)	(0.66–0.76)	(0.67–0.81)
Control (N = 8)	0.61	0.60	0.60	0.62
	(0.57–0.78)	(0.58–0.77)	(0.59–0.70)	(0.59–0.70)
D* (×10^**−3**^ **mm**^**2**^**/s)**				
Treated (N = 14)	2.44	1.43	1.25	1.25
	(1.69–3.09)	(1.30–1.80)	(1.08–1.32)	(1.12–1.50)
Control (N = 8)	2.15	1.95	1.99	1.85
	(1.28–3.08)	(1.25–2.71)	(1.55–2.93)	(1.23–2.82)
***f*p *(%)***				
Treated (N = 14)	17.07	30.59	31.01	35.77
	(13.59–23.99)	(23.73–37.70)	(23.42–39.90)	(25.98–59.65)
Control (N = 8)	22.57	25.12	26.05	31.75
	(12.94–37.54)	(13.43–37.94)	(18.16–39.08)	(21.51–51.38)
**D (×10**^**−3**^ **mm**^**2**^**/s)**				
Treated (N = 14)	0.48	0.39	0.35	0.31
	(0.39–0.51)	(0.27–0.44)	(0.31–0.39)	(0.09–0.40)
Control (N = 8)	0.48	0.50	0.50	0.45
	(0.42–0.51)	(0.42–0.54)	(0.42–0.53)	(0.36–0.49)

**Table 2:** Percentage changes in tumor MRI measurements after treatment compared with pre-treatment.

Median (Interquartile range)	ΔD1 =	ΔD3 =	ΔD7 =
	(D1-D0)/D0*100%	(D3-D0)/D0*100%	(D7-D0)/D0*100%
**ΔTv (%)**			
Treated (N = 14)	8.89	15.00	55.23
	(1.93–29.82)	(25.58–60.52)	(24.95–64.50)
Control (N = 8)	3.29	12.42	130.21
	(1.60–38.07)	(3.33–56.95)	(92.24–338.53)
**ΔADC (%)**			
Treated (N = 14)	3.70	8.19	11.47
	(-3.15–10.79)	(1.31–30.43)	(3.55–38.93)
Control (N = 8)	-0.64	-1.28	1.32
	(-6.52–12.19)	(-5.51–7.51)	(-7.26–8.42)
ΔD* (%)			
Treated (N = 14)	-41.15*	-48.72*	-48.51*
	(-53.56- -21.79)	(-64.91- -28.91)	(-63.61- -30.29)
Control (N = 8)	-9.46	-7.18	-13.94
	(-12.55–1.08)	(-13.08–1.08)	(-21.15–1.40)
**Δ*f*p (%)**			
Treated (N = 14)	79.17*	81.65*	109.53*
	(57.58–90.92)	(50.47–98.89)	(85.03–200,94)
Control (N = 8)	11.28	15.38	40.63
	(-12.37–11.84)	(8.01–55.69)	(28.32–84.94)
**ΔD (%)**			
Treated (N = 14)	-18.90	-26.75*	-35.71*
	(-39.25- -4.06)	(-34.57- -12.12)	(-78.72- -15.17)
Control (N = 8)	5.78	5.43	-6.36
	(-1.45–11.89)	(1.83–18.32)	(-17.24–1.99)

* Significant differences between the treated and control groups at each time point.

### Tumor Volume Measurement

**Fig 3:**
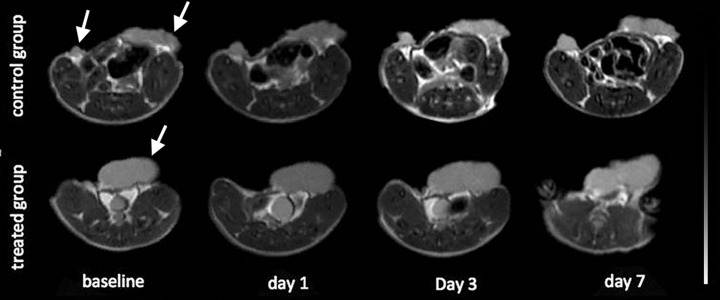
Tumor volume of representative mice for control and treated group. Axial T2W images of a representative mouse in the control group (first row) and a mouse in the treated group (second row, the second tumor of this mouse was out of plane) at time points before (baseline) and after treatment (days 1, 3 and 7). No obvious changes in tumor volume were observed throughout the 7 days post-treatment in both groups.

There were no statistical differences in ΔTv% between the treated and control groups on day 1 (*P* = 0.9141) or day 3 (*P* = 0.7602). Tumor volumes of both groups increased on day 7 compared to days 1 and 3 **(Fig 2A)**; however, no significant difference in ΔTv% between the control and treated groups was observed (*P* = 0.3544). **Fig 3** shows anatomic T2W images and tumor volume changes in two representative mice, one from the control group, and the other from the treated group (The second tumor of this mouse was out of plane). In these two representative animals, no obvious changes in tumor volume were observed throughout the 7 days post-treatment.

### Tumor Diffusion Measurements

ADC values of the treated group continued to increase on days 1, 3 and 7 after treatment, whereas ADC values of the control group were stable **(Fig 1B)**, however, no significant differences between ΔADC_treated_% and ΔADC_control_% were demonstrated on day 1 (*P* = 0.6698), day 3 (*P* = 0.0538) and day 7 (*P* = 0.0686) after treatment **(Fig 2B)**, primarily due to the significant overlap between these two groups.

The fast diffusion parameters D* and *f*p changed in opposite directions with the fast diffusion coefficient (D*) decreasing and fractional volume (*f*p) increasing after treatment **(Fig 1C and 1D)**. D* of the treated group decreased on days 1, 3, and 7, whereas D* of the control group only slightly decreased occurred at the same time points. Significant differences in ΔD* % between the treated and control groups were observed (*P* = 0.0001, *P* = 0.0001 and *P* = 0.001) on days 1, 3 and 7, respectively **(Fig 2C)**. Continuous increases in *f*p of the treated group were noted on days 1 to 7 after treatment, whereas *f*p of the control group did not increase as swiftly after treatment. Significant differences in Δ*f*p% were found between the treated and control groups at each time point (*P* = 0.0001, *P* = 0.0001, and *P* = 0.0007) **(Fig 2D)**.

The slow diffusion parameter D of the treated group continued to decrease on days 1, 3 and 7 after treatment, whereas D values of the control group slightly increased on day 1 but then decreased on day 3 and 7 **(Fig 1E)**. Significant differences between ΔD_treated_% and ΔD_control_% were demonstrated on day 3 (*P* = 0.0133) and day 7 (*P* = 0.0093) after treatment **(Fig 2E)**.

**Fig 4:**
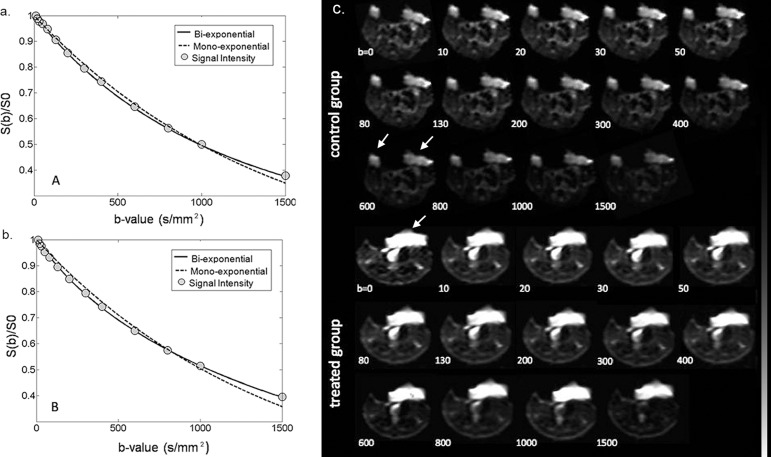
The averaged signal intensity decay and representative IVIM DW images. The averaged signal intensity decay within (a) a treated tumor ROI and (b) a control tumor ROI. Each as a function of a b-value, are plotted on day 1 using both mono-exponential and bi-exponential models. For the treated mouse, D* = 1.02×10^-3^mm^2^/sec, *f*p = 41.6% and ADC = 0.70×10^−3^ mm^2^/sec. For the control mouse, D* = 2.53×10^-3^mm^2^/sec, *f*p = 14.5% and ADC = 0.68×10^−3^ mm^2^/sec. (c) IVIM DW images acquired at increasing b-values in a representative mouse of the treated group and a mouse of the control group.

DW images and signal intensity decay fitting with both mono-exponential and bio-exponential models were demonstrated in two representative animals in the control and treated group (**Fig 4A and 4B**). Within each tumor ROI, DW signal decay was better fitted using the two compartment bi-exponential model than the mono-exponential model. D* was lower and *f*p was higher in the treated tumor when compared with the non-treated tumor, which indicated decreased pseudo-perfusion effect and less distinct separation between fast and slow compartments in tumor tissues secondary to the treatment. Tumor areas were clearly delineated on the images without obvious artifacts **(Fig 4C)**. Compared to the surrounding tissues, tumor areas showed brighter signals due to more restricted tissue water diffusion and T2 shine-though effect.

### Histopathological Characteristics

**Fig 5:**
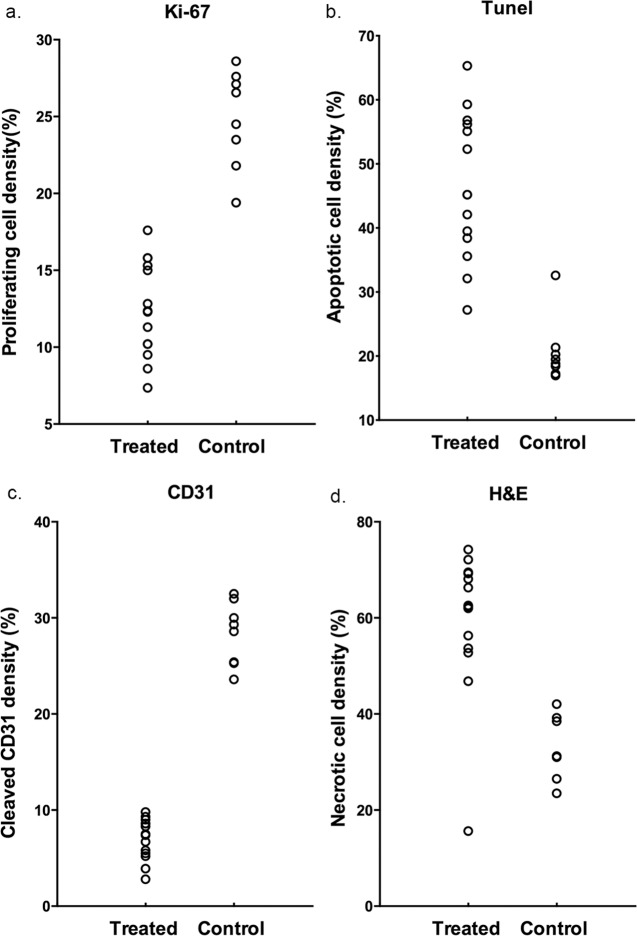
Histopathological analysis of tumors of control and treated group. Histopathological analysis of (a) proliferating cell density (Ki67 staining), (b) apoptotic cell density (TUNEL staining), (c) microvessel density (CD31 staining) and (d) necrosis fraction (H&E staining) of the treated group and control group on day 7 after treatment.

**Fig 6:**
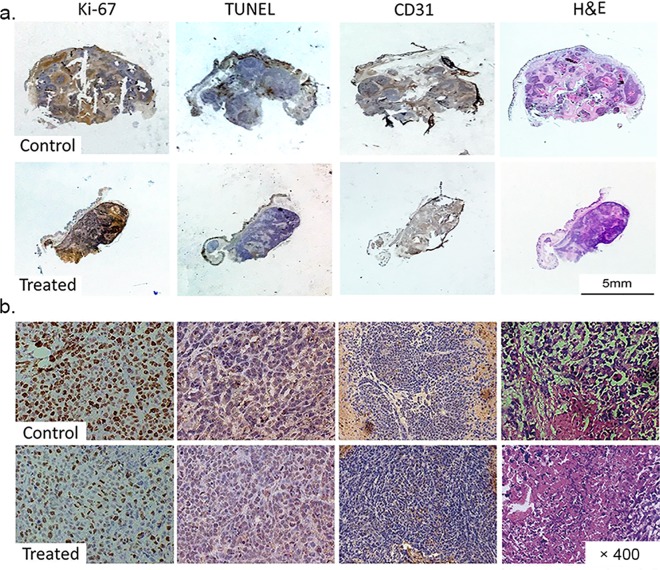
Representative histopathological images of tumor samples on whole mounts and under the microscope (×400). Representative histopathological images of tumor samples (a) on whole mounts and (b) under the microscope (×400) with Ki-67 staining (first column), TUNEL staining (second column), CD31 staining (third column) and H&E staining (last column) of the control group (first and third row) and the treated group (second and fourth row). Brown staining in the Ki-67 images indicates cells in the proliferative state. Brown staining in the TUNEL images indicates cells undergoing apoptosis. Brown staining shown in the CD31 images indicates endothelial cells. H&E images indicate cell necrosis.

The density of proliferating cells identified by Ki-67 staining was lower in the treated group than that in the control group (**Fig 5A**). The apoptotic cell density quantified as the percentage of cells stained positive in TUNEL staining was higher in the treated group than in the control group (**Fig 5B**). Endothelial cell vascularity assessed by CD31 staining demonstrated a trend toward reduced microvessel density after treatment (**Fig 5C**). The tumor necrotic fraction identified by H&E staining increased dramatically after treatment (**Fig 5D**). The median and interquartile ranges of each pathological analysis on day 7 after treatment were listed in **Table 3**. Representative immunohistochemistry images (Ki-67, TUNEL, CD31 and H&E staining) of the whole mount and tumor samples demonstrated different characteristics of tumor tissues between the treated and control groups (**Fig 6**).

**Table 3:** Pathological analysis on day 7 after treatment.

Median(interquartile range)	Ki67 (%)	Tunel (%)	CD31(%)	HE (%)
**Treated (N = 14)**	12.3	45.2	7.45	62.3
	(10.2–15.3)	(38.4–56.2)	(5.55–8.9)	(53.68–69.24)
**Control (N = 8)**	26.83	19.88	27.25	31.1
	(24.25–27.85)	(18.34–24.16)	(24.88–30.5)	(25.75–33.2)
***P***	<0.001	<0.001	<0.001	<0.001

Spearman correlation coefficients between IVIM parameters on each post-treatment time point and end-treatment pathological analyses were listed in **S1 Table**. Post-treatment ADC values had a significant correlation with necrotic fraction. Increasing ADC corresponded with higher necrotic fractions. The highest correlation coefficient between ADC and necrotic fraction (0.611, *P* = 0.003) occurred on day 7. Post-treatment D* had significant correlations with all of the pathological results, with decreased D* corresponding well with decreasing proliferating cell density, increasing apoptotic cell density, decreasing microvessel endothelial cell density and increasing necrotic fraction. High correlation coefficients (>0.6, *P*<0.01) between D* and proliferating cell density and necrotic fraction were observed from day 1 after treatment. In addition, most post-treatment *f*p had significant correlations with all of the pathological characteristics. The higher correlation coefficient (-0.679, *P*<0.01) between *f*p and proliferating cell density was observed on day 1 after treatment. Also, high correlation coefficients (-0.653 to -0.733, *P*<0.01) between *f*p and microvessel endothelial cell density were observed starting from day 1 through day 7 after treatment.

## Discussion

Imatinib (STI571) has antitumor effects on GIST cells by inhibiting cell growth and suppressing vascular endothelial growth factor (VEGF) expression[26]. In the present DWMRI study, based on the two compartmental IVIM model, we found significant changes in fast diffusion coefficient (D*), fractional volume (*f*p), and slow diffusion coefficient (D), as early as one day after Imatinib treatment. In the control group, however, D* and *f*p did not show these changes when compared to the baseline during the course of the study. DWMRI findings correlated well with histopathological changes showing a decrease in proliferating cells and microvessel density and an increase in apoptosis and tumor necrosis in the treated group.

Tumor volume increased in both treated and control groups during the course of the study, but with a smaller volume increase in the treated group on day 7 post-treatment. Increased tumor volumes in the treated group were observed partly due to the formation of intra-tumoral necrosis induced by treatment, which was confirmed by pathological analyses. For some animals in both treated and control groups, no obvious changes in tumor volume were observed throughout the 7 days post-treatment, indicating that tumor volume may not reflect the tumor response to treatment accurately.

In previous clinical studies, ADC values derived from conventional DWMRI have been used to predict responders versus non-responders among GIST patients within one month after Imatinib treatment[8, 15, 16], where increasing ADC values correlated well with the standardized uptake value (SUV) in ^18^F-FDG PET/CT[15, 16]. Higher ADC values of responders compared to non-responders may correlate to the extensive cystic or myxoid degeneration of GIST[8, 27, 28]. In our present study, ADC increased on days 1, 3 and 7 in both treated and control groups with a considerable overlap. Complex interplay between cellularity, the intracellular and extravascular/extracellular spaces, and tissue perfusion[29–31] after treatment may contribute to the overall increase of local water mobility in tumor tissues regardless of treatment; therefore, it is important to evaluate water mobility in different tissue compartments for a more accurate characterization of tissue microstructural properties.

The IVIM DWMRI method, developed by Le Bihan [17, 18, 32], permits characterization of both tissue pseudo-perfusion and diffusion without the need for exogenous contrast agents. At a macroscopic level, the capillary network is distributed in space in a pseudorandom manner, and the overall movement of the blood’s water molecules within the capillaries (i.e. perfusion) mimics the diffusion model. IVIM DWMRI utilizes local water mobility as an endogenous probe for invasive interrogation of tissue microvasculature and microstructure. The perfusion-related fast diffusion coefficient D* is considered proportional to mean capillary segment length and average blood velocity[17].

DWMRI has been exploited to investigate therapeutic responses in different tumor types; however, a variety of results have been reported. In a preclinical study, Joo et al. indicated that D* and *f*p decreased 4 hours after administrating the vascular disrupting agent CKD-516 in a rabbit VX2 liver tumor model, but then both values recovered to baseline at 24 hours after treatment. In several previous clinical studies, increasing *f*p was demonstrated at different time points after treatment whereas the changes in D* had not been observed[22, 25, 33]. *f*p increased two weeks after treatment in advanced hepatocellular carcinoma treated with the antiangiogenic drug sorafenib[25]. In addition, significant increases in *f*p, D, and ADC values 7.5 months after chemotherapy or combined treatment with the targeted medicine cetuximab were demonstrated in responders with squamous cell carcinomas of the head and neck[33]. In another study controversial results were reported: *f*p did not change 2–4 weeks after treatment for rectal cancer, whereas D increased[22]. The variability of IVIM derived measurements in these studies may be attributed to inconsistent IVIM imaging protocols, the range of b-values, and multi-compartment diffusion signal models[17, 29]. In our present study, changes in IVIM derived fast diffusion parameters (decreasing D* and increasing *f*p) were shown as early as 24 hours after initiating Imatinib treatment. Histopathological analyses showed increased cellular apoptosis and decreased microvessel density in response to treatment, both of which contributed to the changes in tissue diffusion properties. As a consequence, the destruction of tumor microvasculature and decreased cellularity led to the mixture of the fast and slow diffusion component after chemotherapy, resulting in a significantly decreased D* and an increased *f*p.

This difference between the fast and slow diffusion coefficient (D* and D) in this study did not reach a magnitude of 10–50, as shown in prior reported studies. Those prior studies attempted to use parameters derived from IVIM model to differentiate malignant and benign breast lesions. Liu, et al. reported that both D (median 0.85 with interquartile range of 0.77 to 0.98×10^-3^mm^2^/sec) and D* (94.71, 70.33 to 113.23×10^-3^mm^2^/sec) were significantly lower compared to surrounding normal tissues[34]. Woo, et al. reported D* of 33.6±15.1×10^−3^ mm^2^/sec and D of 0.91±0.10×10^−3^ mm^2^/sec in hepatocellular carcinomas with poor differentiation[35]. In our study, we found that the signal decay curve did not demonstrate an obviously faster decay at the lower range of b-values compared with the decay at the higher range of b-values, resulting in a lower magnitude of D*. This phenomenon may be attributed to the inherent insufficient vascular supply in GIST xenograft mice model. By comparing the bi-exponential and mono-exponential signal models in signal decay fitting, the bi-exponential model provided better fitting results, which indicated the presence of two compartmental tissue properties in these tumors. Although it is not clear whether the fast diffusion component is completely associated with microvascular perfusion effect, the histolopathological analysis revealed decreasing microvessel density that was related to the decreasing fast diffusion coefficient.

Slow diffusion coefficient (D) is generally considered to be the pure diffusion coefficient describing extracellular, extravascular tissue water mobility [2, 7, 20]. However, we observed significant decreases in D (most values were close to 0) from day 1 after treatment, which is controversial when compared to previous studies [13, 14]. Our explanation for this finding is that D in the bi-exponential model may not represent any real physiological changes but rather may be a covariate parameter in the process of iterative convergence of the nonlinear signal fitting. As discussed above, destruction of the tumor microenvironment after chemotherapy leads to the mixture of two compartments and a deviation from the assumption of bi-exponential signal model. Therefore, the term V_slow_·*e*^-bD^ may reach to a constant term, meaning that D will have to approach zero so that the signal decay may incline to a mono-exponential model. In this situation, resultant D values are not reliable when approaching zero. For a more robust slow diffusion coefficient measurement, we performed the linear fitting of the logarithm DW signal decay with b-value = 200 and 1500 s/mm^2^, where the fast diffusion effect can be ignored. We found that the slow diffusion coefficient derived from the linear fitting of high b-value DW signals increased after treatment (data was not shown in this paper), with a trend similar to the ADC changes.

Our study has several limitations. There are overlaps in various parameters between the control and treated groups, although significant differences in D* and *f*p were detected. These overlaps may be attributed to various baseline measurements and the small simple size. Various baseline (i.e. pre-treatment) measurements were observed, which was due to various degrees of tumor necrosis and heterogeneous inta-tumoral tissue changes during tumor growth in different animals. A relatively small sample size was due to the slow growth rate of GIST cells for the establishment of GIST xenograft tumors, which makes serial pathological analyses at all time points not possible. All our animals were euthanized after imaging at the last time point, and, therefore, pathological analyses were not available along the course of treatment for longitudinal comparisons with imaging features. In addition, tumor ROIs were drawn on the entire tumor areas of the center slice, and the averaged ROI signal intensities were used for IVIM measurements. We chose the whole tumor ROI approach because it was difficult to identify viable tumor tissues only and track them for serial post-treatment measurements in the same animal. Intra-tumoral heterogeneity analyses of IVIM features should be done to provide more information for tumor response evaluation in the future. Lastly, imaging was acquired on a clinical 3T MRI with a dedicated small coil instead of using a high field small animal scanner. The signal-to-noise ratio (SNR) may be limited especially at high b-values. We increased NEX for higher b-values to improve the SNR, which is also a standard parameter setting in clinical DW-MRI protocols. More importantly, implementation and optimization of MRI techniques on a 3T MRI scanner warrants the future translation of these techniques to a study with human subjects and ultimately patients. Overall, this is a pilot study testing the feasibility of IVIM in GIST tumor models for the assessment of tumor response to Imatinib, which, to our knowledge, has not been reported in previous literature. In the future study, multiple treated groups should be given different doses of Imatinib to determine IVIM’s suitability for measuring response. Further technical developments in more advanced diffusion models and comparisons with other MRI techniques such as dynamic contrast enhanced MRI for tumor tissue permeability measurements are warranted.

In conclusion, this study demonstrated that IVIM DWMRI derived fast and slow diffusion parameters provided early and critical information indicating tissue mircostructural and microvasculature changes secondary to the molecular target treatment of GIST with Imatinib. The fast diffusion coefficient D* decreased whereas the fast fractional volume (*f*p) increased throughout the treatment course from as early as one day after treatment. No considerable overlaps were observed in D* and *f*p measurements between the treated and control groups. IVIM fast diffusion measurements have the potential to offer an accurate and early evaluation of Imatinib treatment efficacy, superior to ADC measurement, to facilitate individualized treatment planning and prompt treatment adjustments in future GIST patients.

## Supporting Information

S1 File Relevant data of the study. The sheets named “Tv” “ADC” “DSTARE” “fp” and “D” showed original data of tumor volume and each of the diffusion measurements at each time point, separately. The sheet named “pathology” showed the original data of histopathological analysis, contain proliferating cell density (Ki67 staining), apoptotic cell density (TUNEL staining), microvessel density (CD31 staining) and necrosis fraction (H&E staining) of the two groups.
(XLSX)

S1 Table Spearman coefficients of tumor MRI measurements and pathological analyses. Post-treatment ADC values had a significant correlation with necrotic fraction. Post-treatment D* had significant correlations with all of the pathological results. High correlation coefficients (>0.6, *P*<0.01) between D* and proliferating cell density and necrotic fraction were observed from day 1 after treatment. Most post-treatment *f*p had significant correlations with all of the pathological characteristics.
(DOCX)
