# Correction: The Therapeutic Response of Gastrointestinal Stromal Tumors to Imatinib Treatment Assessed by Intravoxel Incoherent Motion Diffusion-Weighted Magnetic Resonance Imaging with Histopathological Correlation

**DOI:** 10.1371/journal.pone.0174192

**Published:** 2017-03-15

**Authors:** The PLOS ONE Staff

The second author’s name is spelled incorrectly. The correct name is Jie Deng. The correct citation is as follows: Pan F, Deng J, Zhang C, Wang H, Cheng J, Wu W, et al. (2016) The Therapeutic Response of Gastrointestinal Stromal Tumors to Imatinib Treatment Assessed by Intravoxel Incoherent Motion Diffusion-Weighted Magnetic Resonance Imaging with Histopathological Correlation. PLoS ONE 11(12): e0167720. doi:10.1371/journal.pone.0167720. The Publisher apologizes for the error.
