# Supplementary material for: The Therapeutic Response of Gastrointestinal Stromal Tumors to Imatinib Treatment Assessed by Intravoxel Incoherent Motion Diffusion-Weighted Magnetic Resonance Imaging with Histopathological Correlation
Source: PLoS One. 2016 Dec 2;11(12):e0167720. doi: 10.1371/journal.pone.0167720 (PMC5135126; doi:10.1371/journal.pone.0167720)
Supplement: S1 Table — Post-treatment ADC values had a significant correlation with necrotic fraction. Post-treatment D* had significant correlations with all of the pathological results. High correlation coefficients (>0.6, P<0.01) between D* and proliferating cell density and necrotic fraction were observed from day 1 after treatment. Most post-treatment fp had significant correlations with all of the pathological characteristics. (DOCX) [file pone.0167720.s002.docx]

**S1 Table. Spearman coefficients of tumor MRI measurements and pathological analyses**

| **N=22** |  | **Ki67** | **Tunel** | **CD31** | **HE** |
| --- | --- | --- | --- | --- | --- |
| **ADC _D1_** | Coefficient | -0.131 | -0.119 | -0.014 | 0.452* |
|  | Sig. (bilateral) | 0.563 | 0.599 | 0.952 | 0.035 |
| **ADC _D3_** | Coefficient | -0.147 | 0.243 | -0.121 | 0.594** |
|  | Sig. (bilateral) | 0.514 | 0.275 | 0.592 | 0.004 |
| **ADC _D7_** | Coefficient | -0.259 | 0.275 | -0.433* | 0.611** |
|  | Sig. (bilateral) | 0.244 | 0.215 | 0.044 | 0.003 |
| **D* _D1_** | Coefficient | 0.627** | -0.558** | 0.531* | -0.617** |
|  | Sig. (bilateral) | 0.002 | 0.007 | 0.011 | 0.002 |
| **D* _D3_** | Coefficient | 0.656** | -0.508* | 0.569** | -0.657** |
|  | Sig. (bilateral) | 0.001 | 0.016 | 0.006 | 0.001 |
| **D* _D7_** | Coefficient | 0.611** | -0.518* | 0.523* | -0.750** |
|  | Sig. (bilateral) | 0.003 | 0.013 | 0.013 | 0.000 |
| ***f*p _D1_** | Coefficient | -0.679** | 0.542** | -0.674** | 0.574** |
|  | Sig. (bilateral) | 0.001 | 0.009 | 0.001 | 0.005 |
| ***f*p _D3_** | Coefficient | -0.575** | 0.455* | -0.653** | 0.162 |
|  | Sig. (bilateral) | 0.005 | 0.033 | 0.001 | 0.471 |
| ***f*p _D7_** | Coefficient | -0.570** | 0.488* | -0.733** | 0.469* |
|  | Sig. (bilateral) | 0.006 | 0.021 | 0.000 | 0.028 |
| **D _D1_** | Coefficient | 0.380 | -0.367 | 0.659** | -0.156 |
|  | Sig. (bilateral) | 0.081 | 0.093 | 0.001 | 0.488 |
| **D _D3_** | Coefficient | 0.660** | -0.567** | 0.613** | -0.422 |
|  | Sig. (bilateral) | 0.001 | 0.006 | 0.002 | 0.050 |
| **D _D7_** | Coefficient | 0.520* | -0.320 | 0.521* | -0.484* |
|  | Sig. (bilateral) | 0.013 | 0.146 | 0.013 | 0.022 |

**. Significant correlation when confidence (bilateral) is 0.01.

*. Significant correlation when confidence (bilateral) is 0.05.
